# The unique association between the level of plateletcrit and the prevalence of diabetic kidney disease: a cross-sectional study

**DOI:** 10.3389/fendo.2024.1345293

**Published:** 2024-04-25

**Authors:** Shuwu Wei, Xinyu Pan, Yao Xiao, Ruishuang Chen, Junping Wei

**Affiliations:** ^1^ Department of Endocrinology, Guang’anmen Hospital, China Academy of Chinese Medical Sciences, Beijing, China; ^2^ Beijing University of Chinese Medicine, Beijing, China

**Keywords:** diabetic kidney disease, plateletcrit, platelet parameters, type 2 diabetes mellitus, platelet count, inflammation, hemodynamic abnormalities

## Abstract

**Objective:**

The activation of platelets in individuals with type 2 diabetes mellitus (T2DM) triggers inflammation and hemodynamic abnormalities, contributing to the development of diabetic kidney disease (DKD). Despite this, research into the relationship between plateletcrit (PCT) levels and DKD is sparse, with inconsistent conclusions drawn regarding the connection between various platelet parameters and DKD. This highlights the necessity for comprehensive, large-scale population studies. Therefore, our objective is to explore the association between PCT levels and various platelet parameters in relation to DKD.

**Methods:**

In this cross-sectional study, hematological parameter data were collected from a cohort of 4,302 hospitalized Chinese patients. We analyzed the relationships between PCT, platelet count (PLT), mean platelet volume (MPV), platelet distribution width (PDW), platelet large cell ratio (P-LCR), and DKD, along with the urinary albumin-to-creatinine ratio (UACR), and estimated glomerular filtration rate (eGFR). Receiver operating characteristic (ROC) curve analysis was conducted to evaluate the diagnostic potential of these parameters.

**Results:**

DKD patients exhibited significantly higher PCT levels compared to those without DKD. Multivariate regression analysis identified elevated PCT and PLT levels as potential independent risk factors for both DKD and UACR, while lower MPV levels might serve as independent protective factors for eGFR. The areas under the ROC curve for PCT in relation to DKD and UACR (≥30 mg/g) were 0.523 and 0.526, respectively. The area under the ROC curve for PLT in relation to UACR (≥30 mg/g) was 0.523.

**Conclusion:**

PCT demonstrates a weak diagnostic value for T2DM patients at risk of developing DKD and experiencing proteinuria, and PLT shows a similarly modest diagnostic utility for detecting proteinuria. These insights contribute to a deeper understanding of the complex dynamics involved in DKD. Additionally, incorporating these markers into routine clinical assessments could enhance risk stratification, facilitating early interventions and personalized management strategies.

## Introduction

As a chronic, systemic metabolic disease, type 2 diabetes mellitus (T2DM) has emerged as a global health concern, contributing to disability, frailty, and a reduction in life expectancy. In 2021, the crude prevalence of diabetes among individuals aged 20–79 was reported to be 10.5%. Projections indicate an anticipated increase to 11.3% by 2030 and further to 12.2% by 2045. Notably, during this period, the global population is expected to grow by 20%, while the number of individuals with diabetes is estimated to surge by 46% (1). Diabetic kidney disease (DKD), a serious microvascular complication of diabetes, accounts for over 50% of the leading causes of end-stage renal disease (ESRD) globally (2, 3). A substantial portion of DKD, particularly that arising from T2DM, is preventable through early diagnosis and appropriate therapeutic interventions.

The increase in urinary albumin is currently recommended as the clinical biomarker for screening DKD in diabetic patients (4–6). However, its diagnostic value for early-stage DKD identification is limited due to renal injury that precedes albuminuria (7). Consequently, there is a heightened emphasis on finding new biomarkers associated with the onset and progression of DKD at an early stage. Numerous studies have highlighted that chronic inflammation and hemodynamic abnormalities stemming from hyperglycemia and insulin resistance contribute to the deterioration of kidney function in patients with T2DM (8–10). Nonetheless, the assessment of altered levels of inflammatory markers, including interleukin-8, high-sensitivity C-reactive protein, and tumor necrosis factor-alpha (11), along with hemodynamic indices such as fibrinogen (12, 13), has been consistently hampered by cost and technical challenges. These limitations restrict their practical application in daily clinical settings.

The pathophysiology of DKD involves heightened platelet adhesion, activation, and aggregation. These processes are prompted by the dysregulation of various signaling pathways and metabolic disturbances, including insulin resistance, hyperglycemia, and dyslipidemia (14–17). The increased activation of platelets and the elevated release of prothrombotic and proinflammatory agents in diabetes can be attributed to several factors. These include the reduced bioavailability of nitric oxide and the augmented phosphorylation and glycosylation of cellular proteins (18). Existing studies have explored platelet parameters as both cost-effective and easily accessible indicators through a complete blood count. These parameters function as hematological and inflammatory markers linked to DKD. They include platelet count (PLT) (19–21), mean platelet volume (MPV) (21–25), platelet distribution width (PDW) (21, 25), and platelet large cell ratio (P-LCR) (25). However, the findings from these studies exhibit inconsistencies, indicating the need for further research to validate their utility in DKD diagnosis and management.

The plateletcrit (PCT), an underestimated platelet index, serves as an integrated marker derived from the combination of PLT and MPV, calculated as PCT = PLT# × MPV. This composite measure provides more precise information than other platelet indices, offering a comprehensive evaluation of the total platelet mass. It has proven to be an effective screening tool for detecting quantitative abnormalities in platelets. The PCT index has been employed to assess its predictive value of prognosis in various medical conditions, including saphenous vein graft disease (26), polycythemia vera (27), livedoid vasculopathy (28), hepatitis A infection (29), and non-small cell lung cancer (30), among others. Additionally, a close association has been observed between lower PCT levels and compromised peripheral nerve conduction function as well as the presence of neuropathy in individuals with T2DM. This correlation suggests that PCT may serve as a potential biomarker for distal symmetric polyneuropathy (31).

It is noteworthy that there is a scarcity of studies examining the clinical significance of PCT in DKD involving a substantial number of participants. Furthermore, existing research on the association between other platelet parameters and DKD has yielded conflicting results. In this large hospital-based sample study, we endeavor to fill this gap by exploring the relationship between PCT levels, various platelet parameters, and the prevalence of DKD in patients with T2DM.

## Materials and methods

### Study design and participants

A total of 6,306 individuals aged 18 years and older with T2DM admitted to Guang’anmen Hospital from February 2017 to February 2022 were enrolled in the study. Participants who lacked routinely analyzed results for platelet characteristic (*n* = 322) were excluded from the study. Additionally, individuals who had undergone antiplatelet therapy (*n* = 1,682) were also excluded. Consequently, the final number of participants included in the analyses was 4,302. This retrospective study obtained approval from the Medical Ethics Committee of Guang’anmen Hospital, affiliated with the China Academy of Chinese Medical Sciences (approval no. 2023-187-KY), and was conducted in strict adherence to the principles outlined in the Declaration of Helsinki.

### Measurements

Demographic details, blood pressure records, and medical histories were meticulously extracted from the electronic medical records of inpatients by the same trained personnel. Key platelet characteristics, including PLT, MPV, PDW, P-LCR, and PCT, were routinely analyzed using the XS-800i system (Sysmex, Japan). Levels of total cholesterol, triglycerides, high-density lipoprotein (HDL), and low-density lipoprotein (LDL) were measured using the Beckman Coulter AU 680 system (Brea, USA). Hemoglobin A1c (HbA1c) assessments were performed through high-performance liquid chromatography on the MQ-2000PT device (Shanghai, China). Morning urine samples, which were promptly refrigerated, were analyzed for urine albumin and creatinine levels using the Beckman Coulter AU 680 system (Brea, USA). The urine albumin-to-creatinine ratio (UACR) was then calculated. The estimated glomerular filtration rate (eGFR) was determined using the chronic kidney disease epidemiology collaboration equation, specifically designed for individuals of Asian origin. DKD was defined as UACR ≥30 mg/g and/or eGFR <60 mL/min per 1.73 m, following the criteria set by the American Diabetes Association (32).

### Statistical analysis

The current analysis was conducted using IBM SPSS Statistics, Version 26 (IBM Corporation, Armonk, NY, USA). Significance was established at a two-sided *P*-value <0.05. Continuous variables were presented as means ± standard deviation (SD) or medians ± interquartile ranges (IQR), while categorical variables were expressed as percentages (%). For the comparison of continuous and categorical variables, Nonparametric rank sum test or Student’s *t*-test and chi-square test were used, respectively. The PCT levels were divided into quartiles for analysis. Regression analysis was utilized to explore associations between platelet characteristics and DKD, UACR, and eGFR. The results were summarized as odds ratios or beta with 95% confidence intervals (CIs). The cutoff values for PCT and PLT that provided the largest Youden index for predicting DKD or UACR were identified through receiver operating characteristic (ROC) curve analysis.

## Results

The general and sociodemographic characteristics of the study participants are shown in **Table 1**. The analysis ultimately included 4,302 diabetic participants, with a median age of 59 years (interquartile range: 8 years, minimum: 19 years, maximum: 91 years). Among these participants, 2,821 (65.6%) were diagnosed without DKD (non-DKD), while 1,481 (34.4%) were diagnosed with DKD. Compared to non-DKD participants, those with DKD exhibited significantly higher values in several parameters, including age, the number of men, duration of diabetes, HbA1c, total cholesterol, triglycerides, LDL, UACR, systolic blood pressure, and PCT (all with *P*-values <0.05). Although no differences were observed in diastolic blood pressure, PLT, MPV, PDW, and P-LCR between the two groups, HDL and eGFR were significantly lower in DKD patients than in those without DKD (*P* < 0.05).

**Table 1:** General and sociodemographic characteristics of the participants by DKD.

Characteristics	Non-DKD	DKD	*P*-values
*N*	2,821	1,481	–
Age, years	59 ± 14	61 ± 16	<0.001
Men, %	1,525 (63.5%)	876 (68.2%)	0.001
Duration of diabetes, years	14 ± 11	15 ± 12	<0.001
HbA1c, %	8.4 ± 2.5	8.8 ± 2.7	<0.001
Total cholesterol, mmol/L	4.62 ± 1.56	4.73 ± 1.81	0.002
Triglycerides, mmol/L	1.45 ± 1.01	1.77 ± 1.35	<0.001
HDL, mmol/L	1.12 ± 0.35	1.09 ± 0.34	0.001
LDL, mmol/L	2.93 ± 1.17	3.01 ± 1.31	0.009
UACR, mg/g	8.63 ± 8.11	101.86 ± 340.46	<0.001
eGFR, mL/min per 1.73 m^2^	104.00 ± 20.26	88.44 ± 48.54	<0.001
Systolic blood pressure, mmHg	136 ± 19.5	139 ± 20	<0.001
Diastolic blood pressure, mmHg	80 ± 14	80 ± 15	0.722
PCT, ng/mL	0.23 ± 0.07	0.24 ± 0.08	0.014
PLT, × 10^9^/L	224.0 ± 74.0	229.0 ± 79.5	0.057
MPV, fL	10.4 ± 1.2	10.4 ± 1.2	0.479
PDW, fL	12.0 ± 2.6	12.0 ± 2.8	0.943
P-LCR, %	28.2 ± 9.9	28.0 ± 10.4	0.603

The data are presented as means  ±  SD for continuous variables with a normal distribution, medians ± IQR for continuous variables with a skewed distribution, and as numerical proportions for categorical variables. For comparing continuous and categorical variables, Nonparametric rank sum test or Student’s t-test and chi-square test were used, respectively. DKD, diabetic kidney disease; HDL, high-density lipoprotein; LDL, low-density lipoprotein; UACR, urine albumin-to-creatinine ratio; eGFR, estimated glomerular infiltration rate; PCT, plateletcrit; PLT, platelet count; MPV, mean platelet volume; PDW, platelet distribution width; P-LCR, platelet-larger cell ratio.

The characteristics of the participants, categorized by PCT quartiles, are presented in **Table 2**. The participants in the highest PCT quartile, compared to those in the lowest quartile, tended to be younger, had a higher prevalence of females, and exhibited elevated levels of HbA1c, total cholesterol, triglycerides, LDL, and UACR. Additionally, a higher prevalence of DKD and lower eGFR were observed in this group (all *P* for trend <0.05).

**Table 2:** General and sociodemographic characteristics of the participants by PCT quartile levels.

Characteristics	Quartile 0 (≤0.20)	Quartile 1 (>0.20, ≤0.23)	Quartile 2 (>0.23, ≤0.27)	Quartile 3 (>0.27)	*P* for trend
*N*	1247(0.18)	918 (0.22)	1,104 (0.25)	1,033 (0.31)	–
Age, years	62± 13	60 ± 13	58 ± 15	57 ± 17	<0.001
Men, %	874 (70.1%)	565 (61.5%)	556 (50.4%)	406 (39.3%)	<0.001
Duration of diabetes, years	14 ± 11	14 ± 11	14 ± 12	14 ± 12	0.624
HbA1c, %	8.2 ± 2.4	8.4 ± 2.6	8.6 ± 2.7	9.0 ± 2.7	<0.001
Total cholesterol, mmol/L	4.36 ± 1.51	4.62 ± 1.58	4.8 ± 1.61	5.05 ± 1.92	<0.001
Triglycerides, mmol/L	1.45 ± 1.12	1.55 ± 1.21	1.53 ± 1.12	1.8 ± 1.49	<0.001
HDL, mmol/L	1.09 ± 0.34	1.10 ± 0.36	1.13 ± 0.35	1.12 ± 0.34	0.178
LDL, mmol/L	2.73 ± 1.11	2.91 ± 1.20	3.10 ± 1.17	3.14 ± 1.28	<0.001
UACR, mg/g	12.57 ± 33.56	12.76 ± 37.41	12.26 ± 35.51	15.51 ± 54.14	0.001
eGFR, mL/min per 1.73 m^2^	99.85 ± 29.91	99.79 ± 26.35	101.64 ± 26.06	101.74 ± 25.74	0.003
Systolic blood pressure, mmHg	136 ± 21	136 ± 20	138 ± 20	137 ± 20	0.319
Diastolic blood pressure, mmHg	80 ± 13	80 ± 14	81 ± 14	81 ± 15	0.514
DKD, %	416 (33.4%)	298 (32.5%)	366 (33.2%)	401 (38.8%)	0.005

The data are presented as means  ±  SD for continuous variables with a normal distribution, medians ±  interquartile ranges for continuous variables with a skewed distribution, or as numerical proportions for categorical variables. Binary logistic regression analysis was conducted to identify associations between the gender distribution (specifically the number of males) and the prevalence of DKD with PCT quartile levels. Linear regression analysis was utilized to explore associations between age, duration of diabetes, HbA1c, total cholesterol, triglycerides, HDL, LDL, UACR, eGFR, and blood pressure with PCT quartile levels. DKD, diabetic kidney disease; HDL, high-density lipoprotein; LDL, low-density lipoprotein; UACR, urine albumin-to-creatinine ratio; eGFR, estimated glomerular infiltration rate.

We developed various models to assess the independent effects of PCT levels on DKD, UACR, and eGFR. As presented in **Table 3A**, elevated PCT levels were associated with an increased likelihood of DKD, elevated UACR, and reduced eGFR, even after controlling for various confounding variables. These associations remained statistically significant in model 0, which included no adjustments, and in model 1, which included minimal adjustments. The positive correlations between PCT levels and both DKD and UACR were also observed in the fully adjusted model 2. Multifactorial analysis identified PCT as an independent risk factor for DKD and elevated UACR, potentially contributing to a decrease in eGFR.

**Table 3A:** Associations between PCT levels and DKD, UACR, and eGFR.

	PCT levels, ng/mL	
	OR or β (95% CIs)	*P* value
DKD^0^	4.51 (1.53, 13.32)	0.006
DKD^1^	26.03 (8.11, 83.58)	<0.001
DKD^2^	20.54 (5.86, 72.04)	<0.001
UACR^0^	273.67 (109.50, 437.83)	0.001
UACR^1^	439.59 (266.81, 612.36)	<0.001
UACR^2^	398.73 (221.31, 576.15)	<0.001
eGFR^0^	17.65 (4.85, 30.46)	0.007
eGFR^1^	-12.84 (-24.91, -0.76)	0.037
eGFR^2^	-10.42 (-22.96, 2.13)	0.104

Binary logistic regression analysis was performed to identify the relationships between the prevalence of DKD and PCT levels, and the findings were summarized as odds ratios with 95% CIs. Linear regression analysis was utilized to examine the associations between UACR and eGFR with PCT levels, and the results were summarized as beta accompanied by 95% CIs. For the numbers rendered in superscript: 0, the model was not adjusted; 1, the model was adjusted for age, sex, and duration of diabetes; and 2, the model was adjusted for age, sex, duration of diabetes, HbA1c, systolic blood pressure, diastolic blood pressure, total cholesterol, triglycerides, HDL, and LDL. DKD, diabetic kidney disease; UACR, urine albumin-to-creatinine ratio; eGFR, estimated glomerular infiltration rate; PCT, plateletcrit.

We evaluated the associations between PCT quartile levels and DKD, UACR, and eGFR in **Table 3B**. Elevated PCT quartile levels were found to be significantly associated with an increased prevalence of DKD and increased UACR after adjusting for potential confounders. However, following adjustment for potential confounders, no significant associations were observed between PCT quartile levels and eGFR. In comparison to participants in the first quartile of PCT levels, those in the highest quartile exhibited a significant 65% increase in the odds of having DKD after adjustments for age, sex, and duration of diabetes (*P* for trend <0.001). These associations persisted after further adjustments for additional variables, including HbA1c, systolic and diastolic blood pressure, total cholesterol, triglycerides, HDL, and LDL (*P* for trend <0.001). Notably, the participants in the highest PCT quartile showed a significant 52% increase in the prevalence of DKD compared to those in the first quartile. While elevated PCT levels were significantly associated with increased eGFR before any adjustments (*P* for trend = 0.003), no such association was observed after adjusting in model 1 for age, sex, and duration of diabetes (*P* for trend = 0.153) nor in model 2 after adjusting for a comprehensive set of variables (*P* for trend = 0.244). Furthermore, our findings revealed that, compared with the lowest quartile, individuals in the highest quartile exhibited the highest beta for UACR in model 1, and the associations between PCT levels and UACR persisted in model 2 (both *P* for trend <0.001).

**Table 3B:** Associations between PCT quartile levels and DKD, UACR, and eGFR.

	PCT quartile levels, ng/mL				*P* for trend
	Quartile 0	Quartile 1	Quartile 2	Quartile 3	
DKD^0^	Ref.	0.96 (0.80, 1.15)	0.99 (0.83, 1.18)	1.27 (1.07, 1.51)	0.005
UACR^0^	Ref.	14.44 (-12.65, 41.56)	11.88 (-13.87, 37.64)	44.58 (18.36, 70.81)	0.001
eGFR^0^	Ref.	2.01 (-0.10, 4.11)	2.86 (0.85, 4.86)	3.02 (0.98, 5.07)	0.003
DKD^1^	Ref.	1.05 (0.88, 1.27)	1.17 (0.98, 1.40)	1.65 (1.37, 1.98)	<0.001
UACR^1^	Ref.	22.99 (-4.10, 50.08)	28.29 (2.09, 54.49)	68.91 (41.41, 96.42)	<0.001
eGFR^1^	Ref.	0.09 (-1.80, 1.98)	0.01 (-1.82, 1.84)	-1.38 (-3.31, 0.54)	0.153
DKD^2^	Ref.	1.02 (0.84, 1.23)	1.11 (0.92, 1.33)	1.52 (1.25, 1.84)	<0.001
UACR^2^	Ref.	17.05 (-9.46, 43.56)	18.44 (-7.30, 44.18)	57.52 (30.08, 84.96)	<0.001
eGFR^2^	Ref.	0.15 (-1.73, 2.02)	-0.02 (-1.84, 1.80)	-1.13 (-3.07, 0.81)	0.244

Binary logistic regression analysis was performed to identify the relationships between the prevalence of DKD and PCT quartile levels, and the findings were summarized as odds ratios with 95% CIs. Linear regression analysis was utilized to examine the associations between UACR and eGFR with PCT quartile levels, and the results were summarized as beta accompanied by 95% CIs. For the numbers rendered in superscript: 0, the model was not adjusted; 1, the model was adjusted for age, sex, and duration of diabetes; and 2, the model was adjusted for age, sex, duration of diabetes, HbA1c, systolic blood pressure, diastolic blood pressure, total cholesterol, triglycerides, HDL, and LDL. DKD, diabetic kidney disease; UACR, urine albumin-to-creatinine ratio; eGFR, estimated glomerular infiltration rate; PCT, plateletcrit.

**Figure 1:**
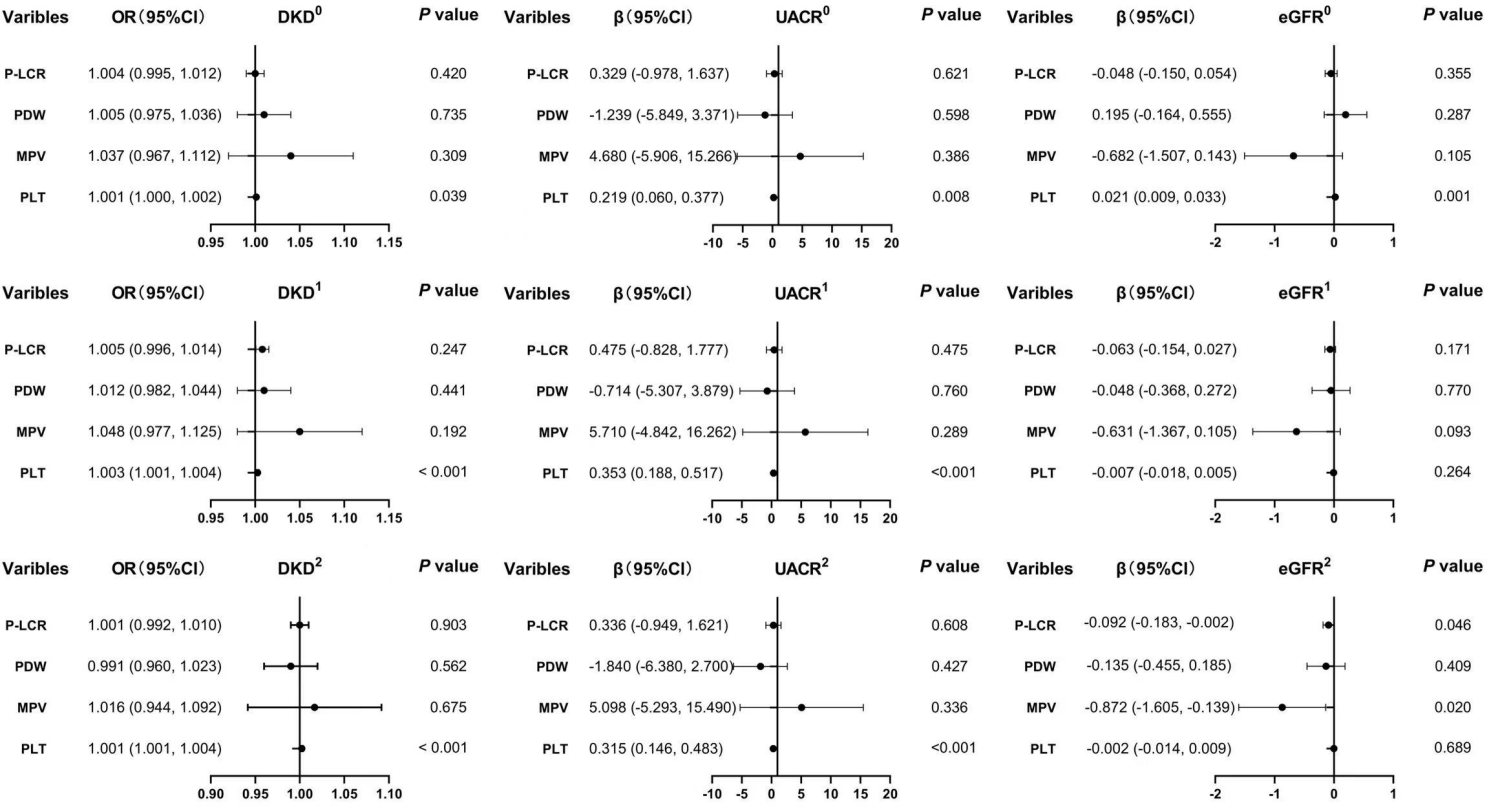
Associations between the levels of PLT, MPV, PDW, P-LCR and DKD, UACE, and eGFR. 0, the model was not adjusted; 1, the model was adjusted for age, sex, and duration of diabetes. 2, the model was adjusted for age, sex, duration of diabetes, HbA1c, systolic blood pressure, diastolic blood pressure, total cholesterol, triglycerides, HDL, and LDL. DKD, diabetic kidney disease; UACR, urine albumin-to-creatinine ratio; eGFR, estimated glomerular infiltration rate; PLT, platelet count; MPV, mean platelet volume; PDW, platelet distribution width; P-LCR, platelet-larger cell ratio.

As illustrated in **Figure 1**, PLT levels exhibit a significant positive correlation with the likelihood of DKD and UACR across three distinct analytical models (model 0, model 1, and model 2). In contrast, PLT levels show a negative correlation with eGFR in the basic model 0. However, no significant associations were found between PCT levels and eGFR in the intermediate model 1 or the fully adjusted model 2. MPV reveals a negative correlation with eGFR in the fully adjusted model 2, while no association was noted in the basic model 0 or the intermediate model 1. Multifactor analysis indicates PLT as an independent risk factor for both DKD and UACR. Additionally, MPV emerges as a potential independent risk factor for eGFR. Notably, no significant associations were found between the prevalence of DKD or the levels of UACR and eGFR with PDW and P-LCR across all models (model 0, model 1, and model 2) (all *P >*0.05).

**Figure 2:**
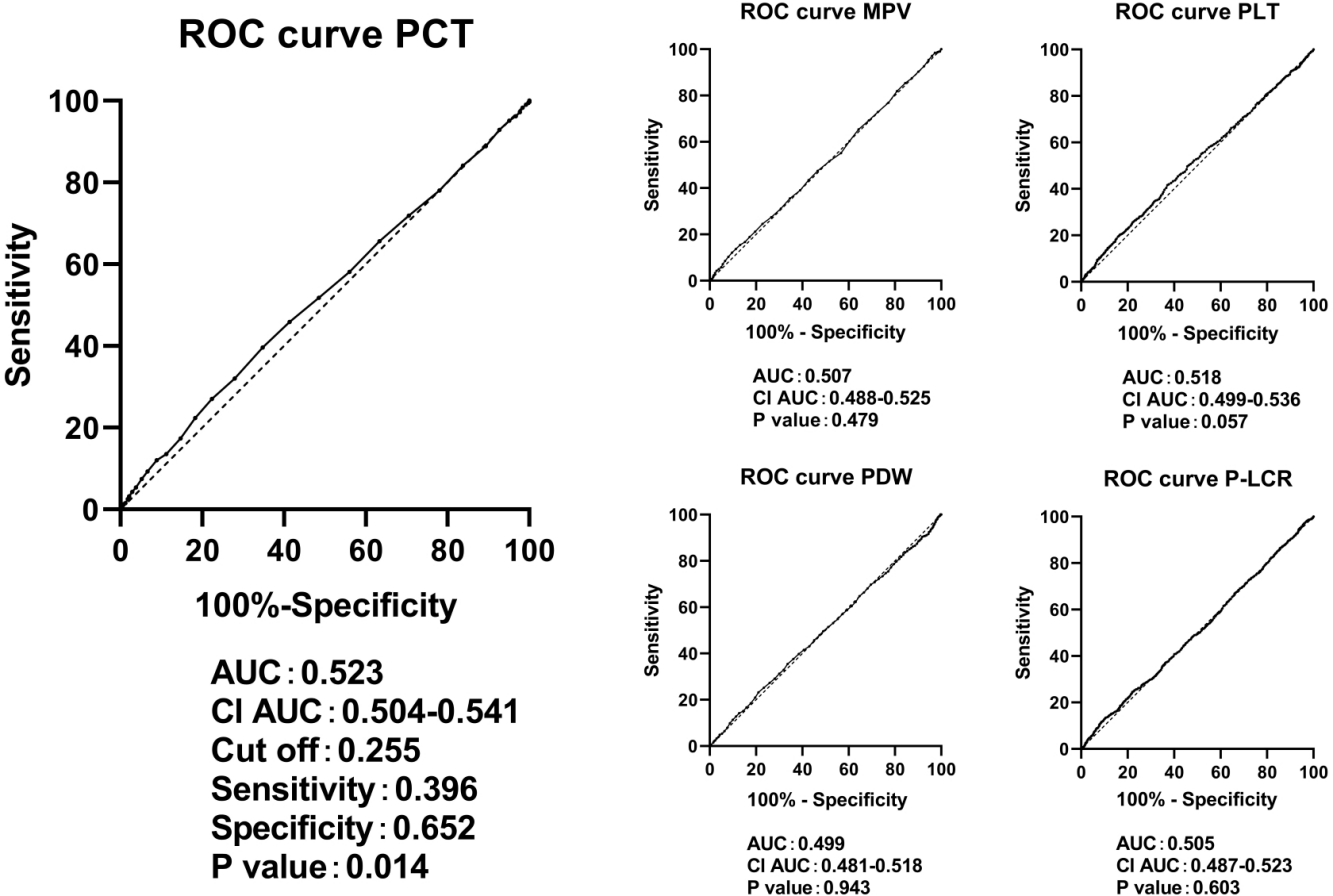
ROC curves in predicting DKD by the levels of PCT, PLT, MPV, PDW, and P-LCR. ROC, receiver operating characteristic; DKD, diabetic kidney disease; PLT, platelet count; MPV, mean platelet volume; PDW; platelet distribution width; P-LCR, platelet-larger cell ratio.

**Figure 2** presents the diagnostic abilities of PCT, PLT, MPV, PDW, and P-LCR for DKD as determined through ROC curve analysis. The area under the ROC curve for PCT levels in predicting DKD was found to be 0.523 (*P* < 0.05). The optimal cutoff point, determined by the highest Youden Index, for PCT was established at 0.255, with a sensitivity of 39.6% and a specificity of 65.2%, suggesting that when PCT levels exceed 0.255, the predictive accuracy for DKD is 0.523. The confidence interval for this prediction spans from 0.504 to 0.541. The true positive rate is 0.396, indicating the proportion of actual DKD cases correctly identified as such, whereas the true negative rate is 0.652, denoting the proportion of non-DKD cases accurately classified.

**Figure 3:**
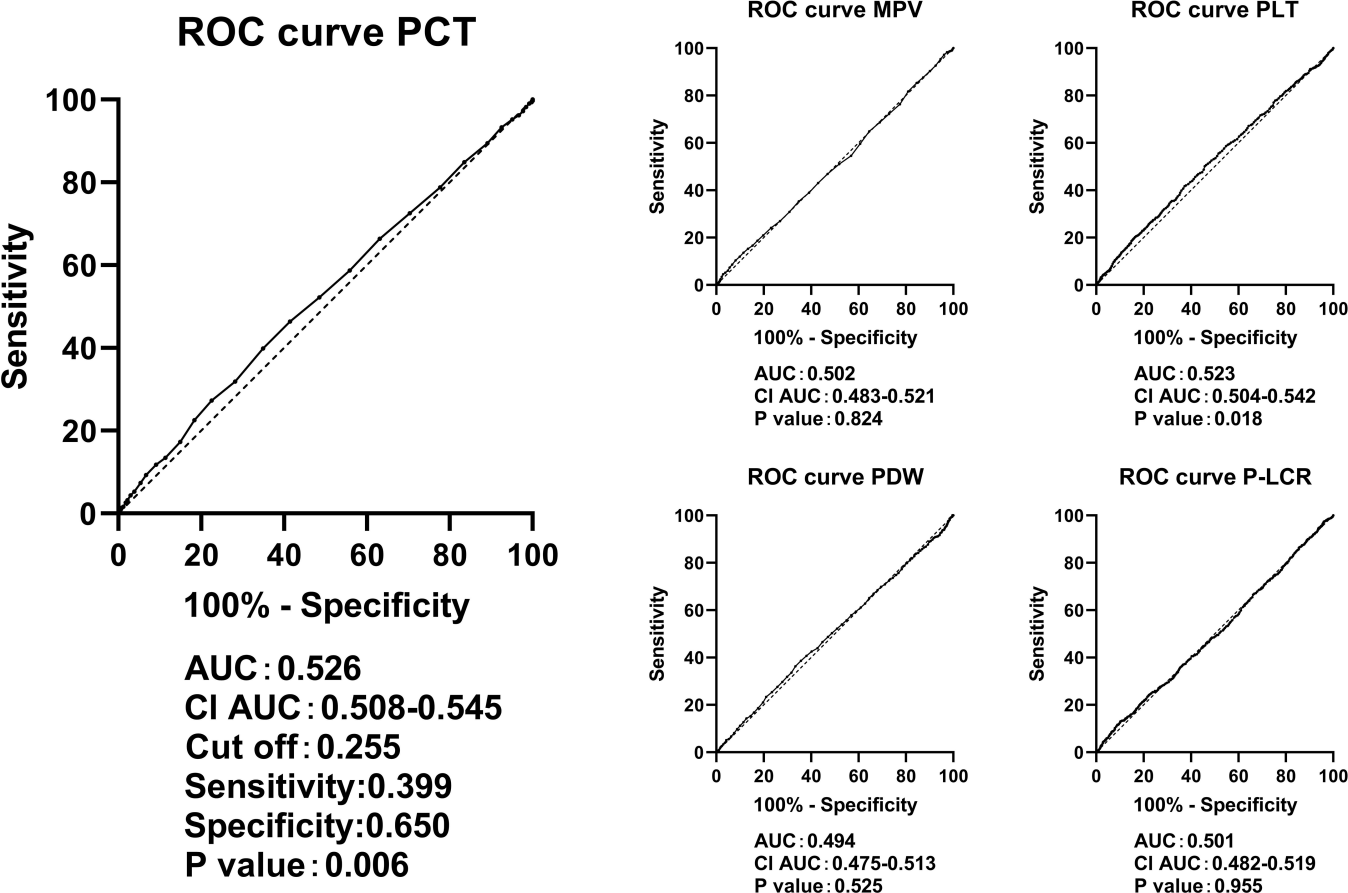
ROC curves in predicting UACR ≥ 30mg/g by the levels of PCT, PLT, MPV, PDW, and P-LCR. ROC, receiver operating characteristic; UACR, urine albumin-to-creatinine ratio; PLT, platelet count; MPV, mean platelet volume; PDW, platelet distribution width; P-LCR, platelet-larger cell ratio.

**Figure 3** shows the diagnostic capabilities of PCT levels for detecting UACR ≥30 mg/g, as assessed through ROC curve analysis. The area under the ROC curve was 0.526 (*P* < 0.05). The cutoff with the biggest Youden index for PCT was 0.255. At this cutoff, the sensitivity was calculated to be 39.9%, and the specificity was 65.0%. Consequently, when PCT levels surpass 0.255, the predictive accuracy for UACR ≥30 mg/g is estimated at 0.526, within a confidence interval of 0.508 to 0.545. The true positive rate is 0.399, while the true negative rate is 0.650. Comparatively, PCT demonstrated a slightly superior diagnostic value for UACR ≥30 mg/g than PLT levels, as the area under the ROC curve for PLT was 0.523. With PLT levels exceeding 241.5, the predictive accuracy for UACR ≥30 mg/g is 0.523, accompanied by a confidence interval ranging from 0.504 to 0.542. The true positive rate is 0.417, while the true negative rate is 0.627.

**Figure 4:**
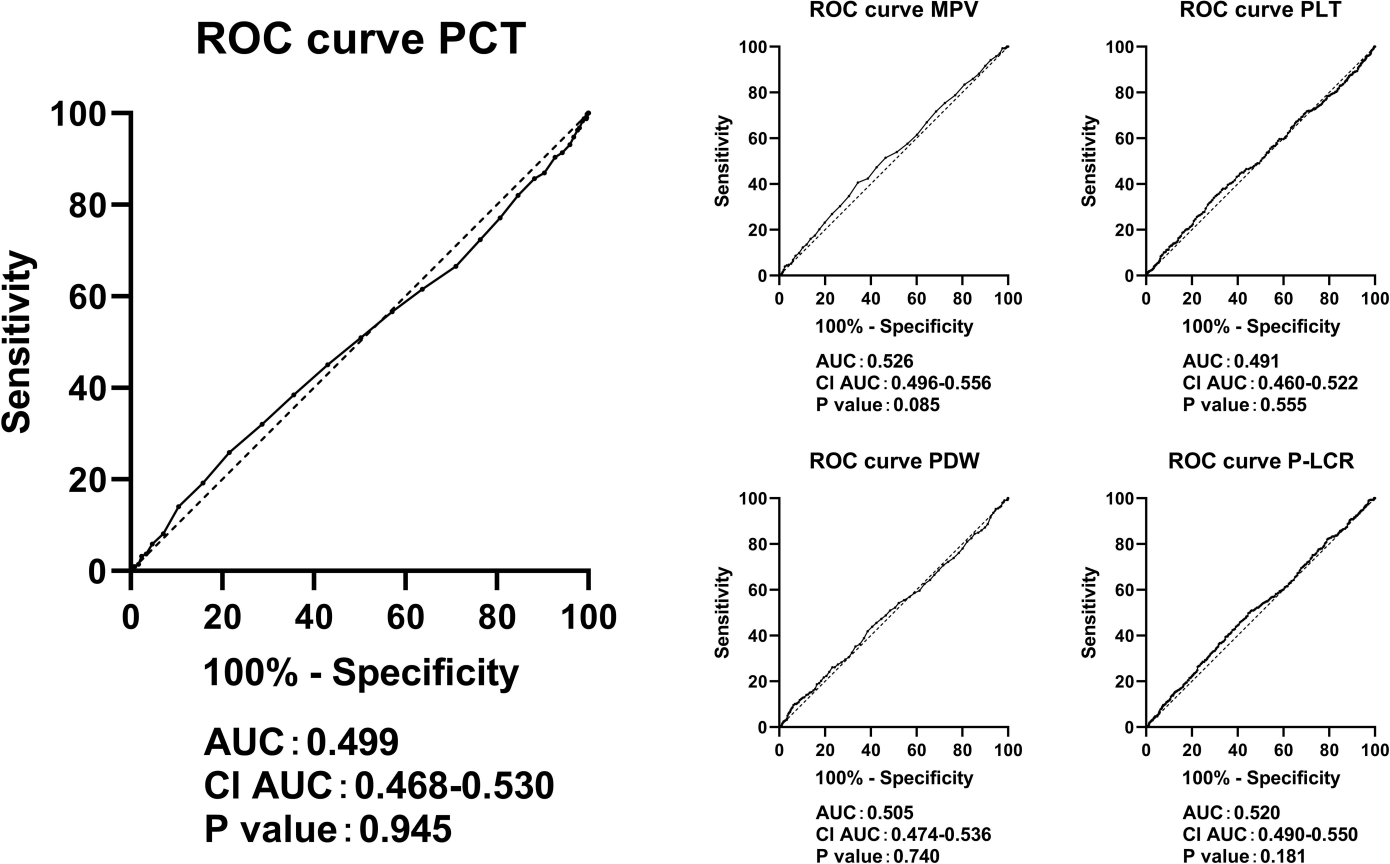
ROC curves in predicting eGFR <60 mL/min per 1.73 m² by the levels of PCT, PLT, MPV, PDW, and P-LCR. ROC, receiver operating characteristic; eGFR, estimated glomerular infiltration rate; PLT, platelet count; MPV, mean platelet volume; PDW, platelet distribution width; P-LCR, platelet-larger cell ratio.

**Figure 4** clearly illustrates that PCT, PLT, MPV, PDW, and P-LCR lacked diagnostic abilities in identifying cases with an eGFR <60 mL/min per 1.73 m².

**Figure 5:**
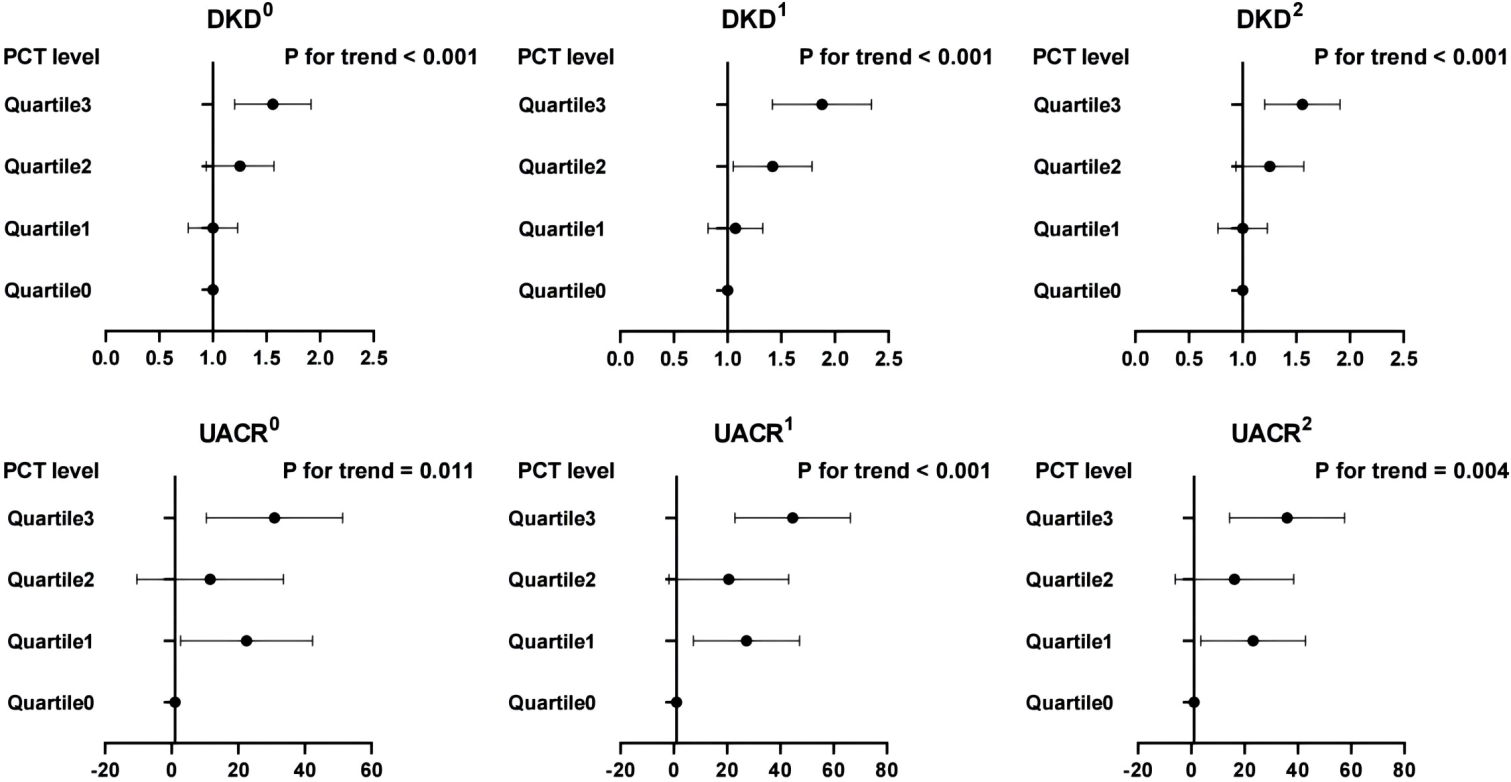
Associations between PCT quartile levels with DKD and UACR in patients with an eGFR ≥ 90 mL/min per 1.73 m^2^. 0, the model was not adjusted; 1, the model was adjusted for age, sex, and duration of diabetes; 2, the model was adjusted for age, sex, duration of diabetes, HbA1c, systolic blood pressure, diastolic blood pressure, total cholesterol, triglycerides, HDL, and LDL. DKD, diabetic kidney disease; UACR, urine albumin-to-creatinine ratio; PCT, plateletcrit.

In **Figure 5**, we delved into the associations between PCT quartile levels and the prevalence of DKD, as well as UACR, specifically in patients with an eGFR ≥90 mL/min per 1.73 m². Our findings indicate a significant association between higher quartiles of PCT levels and an increased occurrence of DKD and elevated UACR, even after controlling for potential confounders. When comparing individuals in the highest quartile of PCT levels to those in the first quartile, the likelihood of having DKD was significantly higher by 84%. This was after adjustments were made for age, sex, and duration of diabetes (*P* for trend <0.001). Further adjustments for a comprehensive set of variables, including age, sex, duration of diabetes, HbA1c, systolic blood pressure, diastolic blood pressure, total cholesterol, triglycerides, HDL, and LDL, confirmed the persistence of these associations. The odds of having DKD in the highest quartile were significantly increased by 53% (*P* for trend <0.001). Additionally, our analysis uncovered that, relative to the lowest quartile, individuals in the highest quartile displayed the greatest beta values for UACR in model 1. This association between elevated PCT levels and increased UACR remained statistically significant in model 2 (both *P* for trend <0.05).

**Figure 6:**
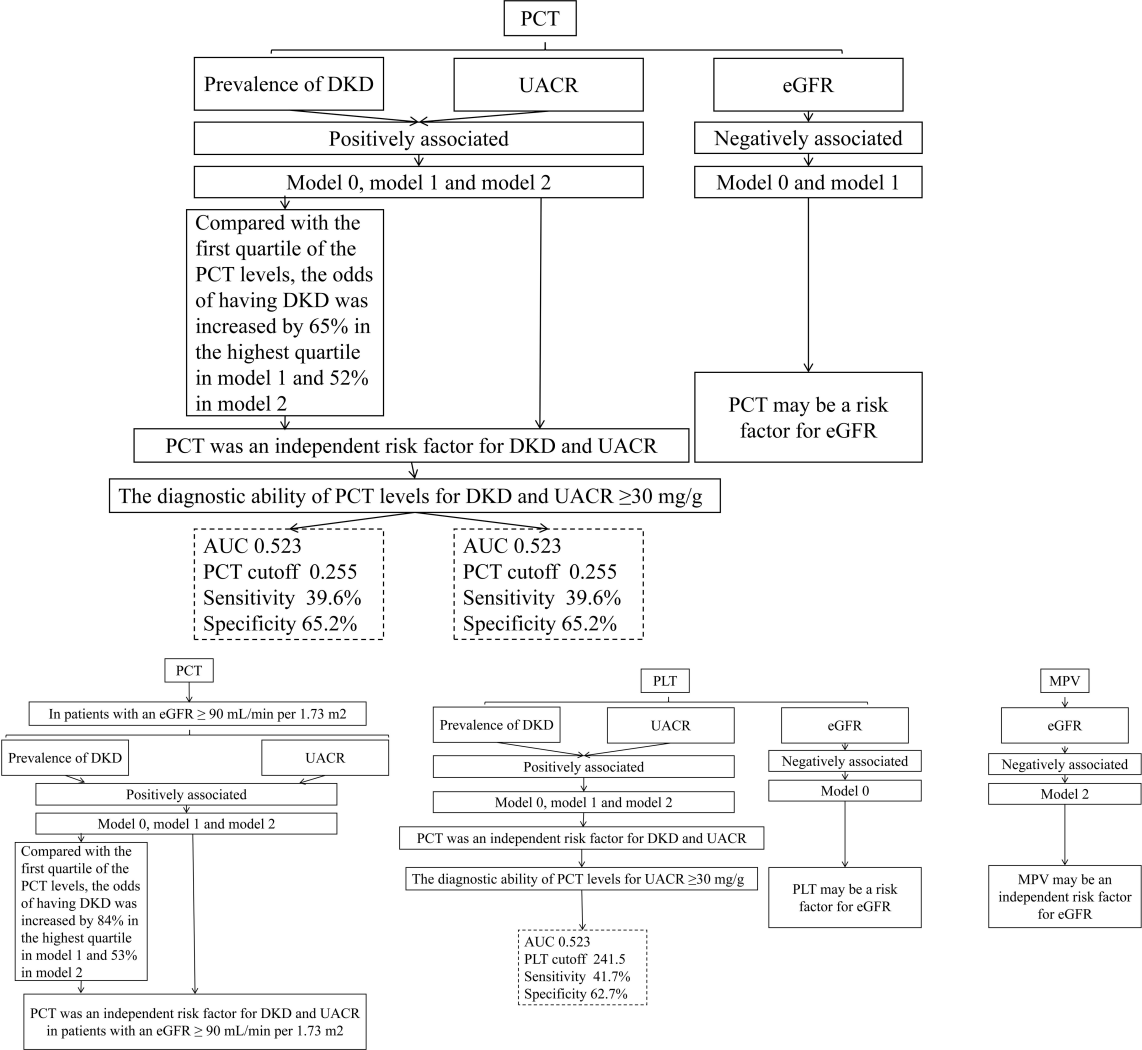
Crucial outcomes of the current research.

**Figure 6** encapsulates the crucial outcomes of our current research.

## Discussion

In individuals with T2DM, insulin resistance may lead to vascular dysfunction characterized by reduced production of prostacyclin and nitric oxide by the vascular endothelium. This reduction can promote increased platelet activity, significantly impacting the disease’s progression (33, 34). Activated platelets, with their dual influence, play a pivotal role in both the development of T2DM and the onset of DKD. They are key contributors to inflammation and hemodynamic abnormalities. The combined effects of systemic and local inflammation, alongside hemodynamic irregularities, are substantial factors in the exacerbation of T2DM and its progression toward DKD. This complex process involves the interaction of numerous bioactive proteins that regulate inflammation and blood flow abnormalities. These proteins are released from the organelles of activated platelets (35, 36). Activated platelets have the capability to produce cytokines or chemokines, such as transforming growth factor-beta. These substances promote not only the production of the extracellular matrix but also its degradation while simultaneously upregulating receptors for matrix interactions. The critical role of these proteins in driving the progression of DKD is well documented (37, 38). Furthermore, the heightened reactivity of platelets is linked to higher levels of procoagulant and tissue factors. This heightened reactivity, combined with the activation of coagulation mechanism and the dysfunction of anticoagulant substances, makes platelets in diabetic patients more likely to adhere to endothelial cells and form aggregates than those in non-diabetic individuals. Such tendencies facilitate thrombus formation, which, in turn, initiates local vascular changes (39, 40). These changes further influence the morphology and functionality of platelets (41). Activated platelets play a significant role in enhancing coagulation function and inflammation, leading to endothelial dysfunction, increased capillary permeability, renal protein leakage, and the presence of albumin in urine. Our research supports these findings, identifying a correlation between heightened platelet activation and the incidence and progression of proteinuria. Previous studies have underscored the significance of increased platelet activation and aggregation as major factors contributing to vascular complications in diabetes (33). Notably, platelet activation has been recognized as a precursor to the onset of microvascular disease in diabetic patients (14). The changes induced by platelet activation, including variations in platelet count, morphology, and function, are observable through parameters such as PCT, PLT, MPV, PDW, and P-LCR.

Platelet parameters are increasingly recognized as novel biomarkers for inflammation and hemodynamic disturbances, valued for their accessibility, cost-effectiveness, minimal invasiveness, and broad applicability in daily clinical practice. Despite their potential, there remains a scarcity of evidence regarding the association between PCT levels and DKD, and the findings concerning the relationship between other platelet parameters (PLT, MPV, PDW, and P-LCR) and DKD are still subject to debate. Thus, it is imperative to explore the relationships between platelet parameters and DKD within the same population. Our research marks a pioneering endeavor in conducting a comprehensive, large-scale population study to investigate the links between PCT levels, other platelet indices, and DKD in humans. The results of our current cross-sectional study reveal distinct associations, showing that patients with DKD exhibited significantly higher PCT levels compared to those without DKD. Furthermore, elevated levels of PCT and PLT were independently correlated with a greater likelihood of DKD and an increased UACR. However, after adjusting for potential baseline confounders, no definitive association was found between PCT or PLT levels and eGFR. Moreover, our analysis indicated a correlation between elevated PCT levels and higher levels of HbA1c and lipids, as detailed in **Table 2**. Based on these findings, we hypothesized that the relationship between PCT levels and eGFR might be modulated by HbA1c and lipid levels. This speculation stems from the detrimental impact of a heightened glucose and lipid milieu in diabetic patients, potentially impacting renal vascular endothelial function and leading to a decline in glomerular filtration function (42). These aligned with our research findings. Our results revealed a positive association between PCT levels and an increased UACR as well as a higher incidence of DKD among participants with a normal eGFR (eGFR ≥90 mL/min per 1.73 m^2^). These results suggest that changes in PCT levels may initiate in T2DM patients exhibiting solely proteinuria. Additionally, the ROC curve analysis suggested that elevated PCT levels could potentially predict the occurrence of albuminuria and DKD more effectively than other platelet-related parameters. Moreover, our findings hinted that an increase in MPV levels may be independently linked to a reduction in eGFR. However, no significant relationships were observed between PDW and P-LCR levels with DKD, UACR, and eGFR. It is noteworthy that PCT, derived from a combination of PLT count and MPV, may provide broader clinical insights than any singular platelet parameter (26). Patients with elevated PCT levels often exhibit an increased platelet count, larger platelet volume, and enhanced platelet activity. The associations between other platelet parameters and DKD seem to be less definitive, potentially due to the fact that individual platelet parameters reflect only the quantity or volume of platelets at a specific level, rendering them less sensitive to changes.

These findings hold significant implications for the clinical management of diabetic patients. The observation of markedly elevated PCT levels in individuals with DKD underscores the potential of PCT as a biomarker for identifying patients at an increased risk of developing complications related to diabetic nephropathy. Multivariate analysis further emphasizes the independent association between elevated levels of PCT and PLT with both DKD and an increased UACR, providing clinicians valuable insights into identifying potential risk factors. Moreover, the ROC curve analyses suggest that PCT and PLT levels can serve as discriminative markers for DKD and elevated UACR. Incorporating these biomarkers into routine clinical evaluations could improve risk stratification processes, facilitating early intervention and the adoption of personalized management strategies for patients. The modest yet noticeable predictive accuracy observed in the ROC curves merits further exploration and potential validation in larger cohorts. These insights contribute to an enhanced understanding of the intricate dynamics associated with DKD. As we improve our predictive capabilities and identify at-risk individuals, opportunities for customized therapeutic strategies and preventive interventions become apparent. These could include intensified monitoring or lifestyle modifications aimed at slowing the progression of kidney disease. This research represents a significant step toward bridging the gap between predictive modeling and its clinical application, thereby enriching our approach to managing complications associated with diabetes.

Our research was conducted on a large scale involving hospitalized patients, which ensured a substantial sample size and enabled rigorous control over participant quality. To yield more reliable results, we meticulously adjusted for confounding factors. Nevertheless, as a single-center cross-sectional study, it does not have the capability to establish causal relationships. Although a correlation between PCT and DKD was observed, caution should be exercised in interpreting these findings. Prospective studies are essential to confirm whether PCT can be considered an independent risk factor. The study included a relatively small number of patients with eGFR <60 mL/min per 1.73 m^2^, which may restrict the generalizability of the current results to patients diagnosed with DKD based on eGFR criteria. A noteworthy limitation was the absence of body mass index (BMI) data for participants, preventing BMI from being included in the analysis. Given BMI’s known associations with insulin resistance and dyslipidemia—factors that can influence the risk and progression of DKD—the lack of BMI as a confounding variable may hinder our understanding of the relationships between platelet indices and DKD. Future research endeavors should consider including BMI data and stratifying analysis based on BMI. This approach may reveal relationships between platelet parameters and DKD that were not apparent due to the absence of BMI data.

## Conclusion

Upon adjusting for potential confounders, our analysis revealed a significant correlation between elevated levels of PCT and PLT with an increased likelihood of DKD and the occurrence of albuminuria. Additionally, PCT was found to have a modest diagnostic value for DKD and UACR ≥30 mg/g. These findings suggest that monitoring PCT levels, which is a straightforward, cost-effective, and widely available method, could prove advantageous for the early screening of DKD. Nonetheless, to establish a causal relationship between PCT levels and DKD, additional cohort studies are imperative.

## Data availability statement

The raw data supporting the conclusions of this article will be made available by the authors, without undue reservation.

## Ethics statement

The studies involving humans were approved by Ethical committee in Guanganmen Hospital, China Academy of Chinese Medical Sciences (Approval No. 2023-187-KY). The studies were conducted in accordance with the local legislation and institutional requirements. The participants provided their written informed consent to participate in this study.

## Author contributions

SW: Conceptualization, Data curation, Formal analysis, Methodology, Writing – original draft, Writing – review & editing, Investigation, Software. XP: Data curation, Investigation, Resources, Writing – original draft. YX: Data curation, Investigation, Resources, Writing – original draft. RC: Data curation, Investigation, Resources, Writing – original draft. JW: Funding acquisition, Project administration, Software, Supervision, Validation, Visualization, Writing – review & editing.

## References

[1] International Diabetes Federation. IDF diabetes atlas. 10th edn. Brussels, Belgium: International Diabetes Federation (2021).

[2] Afkarian M Zelnick LR Hall YN Heagerty PJ Tuttle K Weiss NS. Clinical manifestations of kidney disease among US adults with diabetes, 1988-2014. JAMA. (2016) 9316:602–10. doi: 10.1001/jama.2016.10924 PMC5444809 27532915

[3] Martínez-Castelao A Navarro-González JF Górriz JL de Alvaro F. The concept and the epidemiology of diabetic nephropathy have changed in recent years. J Clin Med. (2015) 4:1207–16. doi: 10.3390/jcm4061207 PMC4484995 26239554

[4] Pugliese G Penno G Natali A Barutta F Di Paolo S Reboldi G. Diabetic kidney disease: new clinical and therapeutic issues. Joint position statement of the Italian Diabetes Society and the Italian Society of Nephrology on “The natural history of diabetic kidney disease and treatment of hyperglycemia in patients with type 2 diabetes and impaired renal function”. J Nephrol. (2020) 33:9–35. doi: 10.1007/s40620-019-00650-x 31576500 PMC7007429

[5] Gluhovschi C Gluhovschi G Petrica L Timar R Velciov S Ionita I. Urinary biomarkers in the assessment of early diabetic nephropathy. J Diabetes Res. (2016) 2016:4626125. doi: 10.1155/2016/4626125 27413755 PMC4927990

[6] Winter L Wong LA Jerums G Seah JM Clarke M Tan SM. Use of readily accessible inflammatory markers to predict diabetic kidney disease. Front Endocrinol (Lausanne). (2018) 9:225. doi: 10.3389/fendo.2018.00225 29910771 PMC5992400

[7] AKh L. Diabetic nephropathy - complications and treatment. Int J Nephrol Renovasc Dis. (2014) 7:361–81. doi: 10.2147/IJNRD.S40172 PMC4206379 25342915

[8] Ito M Gurumani MZ Merscher S Fornoni A. Glucose- and non-glucose-induced mitochondrial dysfunction in diabetic kidney disease. Biomolecules. (2022) 12:351. doi: 10.3390/biom12030351 35327540 PMC8945149

[9] Ferrucci L Fabbri E. Inflammageing: chronic inflammation in ageing, cardiovascular disease, and frailty. Nat Rev Cardiol. (2018) 15:505–22. doi: 10.1038/s41569-018-0064-2 PMC6146930 30065258

[10] Fujita T Hemmi S Kajiwara M Yabuki M Fuke Y Satomura A. Complement-mediated chronic inflammation is associated with diabetic microvascular complication. Diabetes Metab Res Rev. (2013) 29:220–6. doi: 10.1002/dmrr.2380 23280928

[11] Uwaezuoke SN. The role of novel biomarkers in predicting diabetic nephropathy: a review. Int J Nephrol Renovasc Dis. (2017) 10:221–31. doi: 10.2147/IJNRD PMC5566367 28860837

[12] Lee S Lee MY Nam JS Kang S Park JS Shin S. Hemorheological approach for early detection of chronic kidney disease and diabetic nephropathy in type 2 diabetes. Diabetes Technol Ther. (2015) 17:808–15. doi: 10.1089/dia.2014.0295 26214546

[13] Zhang J Wang Y Zhang R Li H Han Q Wu Y. Serum fibrinogen predicts diabetic ESRD in patients with type 2 diabetes mellitus. Diabetes Res Clin Pract. (2018) 141:1–9. doi: 10.1016/j.diabres.2018.04.025 29684616

[14] Hendra TJ Yudkin JS. ‘Spontaneous’ platelet aggregation in whole blood in diabetic patients with and without microvascular disease. Diabetes Med. (1992) 9:247–51. doi: 10.1111/j.1464-5491.1992.tb01770.x 1576806

[15] Ferreiro JL JA Gómez-Hospital Angiolillo DJ. Platelet abnormalities in diabetes mellitus. Diabetes Vasc Dis Res. (2010) 7:251–9. doi: 10.1177/1479164110383994 20921090

[16] Kim JH HY B Kim SY. Response: clinical marker of platelet hyperreactivity in diabetes mellitus (diabetes metab j 2013;37:423-8). Diabetes Metab J. (2014) 38:160–1. doi: 10.4093/dmj.2014.38.2.160 PMC4021304 24851211

[17] Suslova TE Sitozhevskii AV Ogurkova ON Kravchenko ES Kologrivova IV Anfinogenova Y. Platelet hemostasis in patients with metabolic syndrome and type 2 diabetes mellitus: cGMP- and NO-dependent mechanisms in the insulin-mediated platelet aggregation. Front Physiol. (2015) 5:501. doi: 10.3389/fphys.2014.00501 25601838 PMC4283519

[18] El Haouari M Rosado JA. Platelet signalling abnormalities in patients with type 2 diabetes mellitus: a review. Blood Cells Mol Dis. (2008) 41:119–23. doi: 10.1016/j.bcmd.2008.02.010 18387322

[19] Pan L Ye Y Wo M Bao D Zhu F Cheng M. Clinical significance of hemostatic parameters in the prediction for type 2 diabetes mellitus and diabetic nephropathy. Dis Markers. (2018) 2018:5214376. doi: 10.1155/2018/5214376 29511389 PMC5817264

[20] Sterner G Carlson J Ekberg G. Raised platelet levels in diabetes mellitus complicated with nephropathy. J Intern Med. (1998) 244:437–41. doi: 10.1046/j.1365-2796.1998.00349.x 9893096

[21] Buch A Kaur S Nair R Buch A Kaur S Nair R Jain A. Platelet volume indices as predictive biomarkers for diabetic complications in Type 2 diabetic patients. J Lab Physicians. (2017) 9:84–8. doi: 10.4103/0974-2727.199625 PMC5320886 28367021

[22] Turgutalp K Özhan O Akbay E Tombak A Tiftik N Ozcan T. Mean platelet volume and related factors in patients at different stages of diabetic nephropathy: a preliminary study. Clin Appl Thromb Hemost. (2014) 20:190–5. doi: 10.1177/1076029612456734 22914809

[23] Ünübol M Ayhan M Güney E. The relationship between mean platelet volume with microalbuminuria and glycemic control in patients with type II diabetes mellitus. Platelets. (2012) 23:475–80. doi: 10.3109/09537104.2011.634934 22122310

[24] Hekimsoy Z Payzin B Ornek T Kandoğan G. Mean platelet volume in Type 2 diabetic patients. J Diabetes Complications. (2004) 18:173–6. doi: 10.1016/S1056-8727(02)00282-9 15145330

[25] Jindal S Gupta S Gupta R Kakkar A Singh HV Gupta K. Platelet indices in diabetes mellitus: indicators of diabetic microvascular complications. Hematology. (2011) 16:86–9. doi: 10.1179/102453311X12902908412110 21418738

[26] Akpinar I Sayin MR Gursoy YC Karabag T Kucuk E Buyukuysal MC. Plateletcrit. A platelet marker associated with saphenous vein graft disease. Herz. (2014) 39:142–8. doi: 10.1007/s00059-013-3798-y 23575980

[27] Krečak I Zekanović I Morić Perić M Holik H Coha B Periša V. High plateletcrit may be associated with thrombotic risk in polycythemia vera. Int J Lab Hematol. (2023) 45:799–801. doi: 10.1111/ijlh.14073 37050866

[28] Gao Y Jin H. Platelet count and plateletcrit: Potential haematological biomarkers for livedoid vasculopathy? Australas J Dermatol. (2022) 63:e200–5. doi: 10.1111/ajd.13884 35635484

[29] Coskun ME Alidris A Temel MT Akbayram S Hizli S. Plateletcrit: A possible biomarker of inflammation in hepatitis A infection. Niger J Clin Pract. (2019) 22:727–30. doi: 10.4103/njcp.njcp_331_18 31089030

[30] Hur JY Lee HY Chang HJ Choi CW Kim DH Eo WK. Preoperative plateletcrit is a prognostic biomarker for survival in patients with non-small cell lung cancer. J Cancer. (2020) 11:2800–7. doi: 10.7150/jca.41122 PMC7086273 32226498

[31] Qian Y Zeng Y Lin Q Huang H Zhang W Yu H. Association of platelet count and plateletcrit with nerve conduction function and peripheral neuropathy in patients with type 2 diabetes mellitus. J Diabetes Investig. (2021) 12:1835–44. doi: 10.1111/jdi.13535 PMC8504918 33650778

[32] American Diabetes Association. 11. Microvascular complications and foot care: standards of medical care in diabetes-2019. Diabetes Care. (2019) 42:S124–38. doi: 10.2337/dc19-S011 30559237

[33] Vinik AI Erbas T Park TS Nolan R Pittenger GL. Platelet dysfunction in type 2 diabetes. Diabetes Care. (2001) 24:1476–85. doi: 10.2337/diacare.24.8.1476 11473089

[34] Honing ML Morrison PJ Banga JD Stroes ES Rabelink TJ. Nitric oxide availability in diabetes mellitus. Diabetes Metab Rev. (1998) 14:241–9. doi: 10.1002/(ISSN)1099-0895 9816472

[35] Hagita S Osaka M Shimokado K Yoshida M. Adipose inflammation initiates recruitment of leukocytes to mouse femoral artery: role of adipo-vascular axis in chronic inflammation. PloS One. (2011) 6:e19871. doi: 10.1371/journal.pone.0019871 21625491 PMC3098847

[36] Ripoche J. Blood platelets and inflammation: their relationship with liver and digestive diseases. Clin Res Hepatol Gastroenterol. (2011) 35:353–7. doi: 10.1016/j.clinre.2011.02.012 21482218

[37] Sharma K McGowan TA. TGF-beta in diabetic kidney disease: role of novel signaling pathways. Cytokine Growth Factor Rev. (2000) 11:115–23. doi: 10.1016/S1359-6101(99)00035-0 10708959

[38] Ghoshal K Bhattacharyya M. Overview of platelet physiology: its hemostatic and nonhemostatic role in disease pathogenesis. ScientificWorldJournal. (2014) 2014:781857. doi: 10.1155/2014/781857 24729754 PMC3960550

[39] Picard F Adjedj J Varenne O. Le diabète, une pathologie prothrombotique [Diabetes Mellitus, a prothrombotic disease]. Ann Cardiol Angeiol (Paris). (2017) 66:385–92. doi: 10.1016/j.ancard.2017.10.011 29106832

[40] Kaur R Kaur M Singh J. Endothelial dysfunction and platelet hyperactivity in type 2 diabetes mellitus: molecular insights and therapeutic strategies. Cardiovasc Diabetol. (2018) 17:121. doi: 10.1186/s12933-018-0763-3 30170601 PMC6117983

[41] Vallon V Thomson SC. The tubular hypothesis of nephron filtration and diabetic kidney disease. Nat Rev Nephrol. (2020) 16:317–36. doi: 10.1038/s41581-020-0256-y PMC7242158 32152499

[42] Thomas MR Storey RF. The role of platelets in inflammation. Thromb Haemost. (2015) 114:449–58. doi: 10.1160/TH14-12-1067 26293514

